# Contribution to the diagnosis of stenosis of the aortic isthmus

**DOI:** 10.1111/j.0954-6820.1948.tb12031.x

**Published:** 2009-04-24

**Authors:** I G Porjé

**Affiliations:** Stockholm

## Discussion

### Tillgren

One may ask what purpose is served by introducing a new sign into a disease that can be so well diagnosed clinically as isthmus stenosis. Firstly, then, it is always valuable to have a pathognomonic symptom which can be registered without discomfort to the patient – that is to say, in the form of pulse curves. Secondly, of course, these cases have a great value of their own as material for pulse-wave studies.

### Lindquist

Does the diagnostic method, really allow of the conclusion that thestenosis situated in the isthmus aorta? After all, the aorta contains obstructions inother places and of other kinds.

